# P/CVE-as-counterinsurgency: Police violence and police reform in Kenya’s counterterrorism agenda

**DOI:** 10.1177/09670106241301672

**Published:** 2025-02-14

**Authors:** Elizabeth Mesok, Darja Schildknecht

**Affiliations:** University of Basel Faculty of Humanities and Social Sciences, Switzerland; University of Basel Faculty of Humanities and Social Sciences, Switzerland

**Keywords:** Counterinsurgency, counterterrorism, Kenya, police, security, violent extremism

## Abstract

This article considers the calls for police reform and the continuation of police brutality to be twinning modes of policing within Kenya’s broader counterterrorism and preventing and countering violent extremism (P/CVE) architecture. Rather than seeing ongoing police brutality as a failure of, or at odds with, calls for police reform, we argue that what appears to be a paradox is actually indicative of a dialectic central to civil counterinsurgency – a dialectic comprising what we call ‘coercive compliance’ and ‘abject coercion’. Based on extensive field research in Kenya, this article centers the institution of the police as an integral mode of P/CVE-as-counterinsurgency to analyze various manifestations of police power, including international compliance vis-a-vis police reform, police brutality, and community engagement. We further theorize P/CVE-as-counterinsurgency in conversation with Kenya’s colonial history, arguing that police violence is neither new nor unique to today’s counterterrorism regime. Rather, we argue that contemporary policing in Kenya contains sediment of racialized counterinsurgency tactics that were critical to colonial rule in the mid-20th century. In conclusion, we offer reflections on other security contexts where the dialectic of coercive compliance and abject coercion might serve as a useful analytic.

## Introduction

In January 2022, dozens of bodies floated to the shores of the Yala River in Western Kenya. The bodies, all men, showed signs of torture and drowning; many had their hands bound behind their backs or their heads wrapped in plastic bags. Police claim they have launched an investigation, while human rights activists say it is the police that are responsible (Kimari and Glück, 2023). In Kenya, assuming the police are culpable for suspicious deaths stems from a long history of police brutality, corruption, and impunity. In 2021, the coalition Missing Voices, composed of multiple human rights organizations, documented 219 cases of police killings and enforced disappearances. The majority of victims are men, although there has been a noted increase in women victims in recent years, and are overwhelmingly from poor neighborhoods of Nairobi and Mombasa (Njoki and Gachihi, 2020). In Mathare, the second-largest informal settlement in Kenya located in the Eastlands of Nairobi, police were accused of extrajudicial killings 11 out of 12 months in 2021. Despite the global emergence of the preventing and countering violent extremism (P/CVE) agenda, premised on the preventative measures of good governance (which necessarily includes police reform), peacebuilding, and development, police brutality in the name of the prevention of terrorism continues unabated (Attree, 2017; Okia, 2011; Ometo, 2020; Vinson, 2015).

In this article, we argue that the calls for police reform *and* the persistent issue of police brutality are *both* technologies embedded within a broader counterterrorism and P/CVE architecture. Rather than seeing ongoing police brutality as a failure of, or at odds with, calls for police reform, we argue that what appears to be a paradox is actually a dialectic central to civil counterinsurgency. In recent scholarship, we theorized the policy and practice of P/CVE as commensurate with the tactics of civil counterinsurgency and as enacted through ‘police power’ – or, the discursive power wielded by the development and peacebuilding sectors to compel populations to participate in their own security and the material power enacted by police forces (Mesok, 2022). In this article, we build on a theorization of P/CVE-as-counterinsurgency, by which we mean that P/CVE, through formal institutions including but not limited to the police, works through a dialectic of violence and social engagement, ensuring governability and otherwise marking for elimination those deemed threats to the social and political order. Here, we follow scholarship that theorizes and historicizes counterinsurgency as a mode of governance that requires both military action and the management of social and political thought (Charbonneau, 2019, 2021; Kienscherf, 2016; Schrader, 2019). Naming P/CVE *as* counterinsurgency thus situates P/CVE and counterterrorism more broadly within a longer tradition of pacification, which requires the dialectic of ‘violent containment and liquidation . . . [and] a pastoral biopolitics of social improvement’ (Schrader, 2019: 48). This article analyzes the various manifestations of police power both required and produced by such a dialectic, including international compliance vis-a-vis police reform, police brutality, and community engagement.

While recent scholarship has focused on the expansion of policing within P/CVE (Mesok, 2022; Thompson, 2023), there has yet to be an analysis of the role of police in P/CVE within a conceptual framework that specifically understands P/CVE as a mode of counterinsurgency. By doing this, we depart from literature on P/CVE that tends to focus more on best practices or lessons learned (Brett, 2023; Fransen, 2023; Koehler et al., 2023) or on assessing the implementation and effectiveness of P/CVE programming (Shanaah, 2023; Shanaah and Heath-Kelly, 2022; Williams, 2022). Rather, we are in conversation with critical security scholarship that analyzes P/CVE as a form of governance wielded by the Global North to police and discipline populations in and of the Global South through conceptualizations of political violence that are deeply racialized (Aziz, 2017; Griffin and Khalid, 2022; Nguyen, 2023; Martini 2021; Shepherd, 2022). Our understanding of P/CVE-as-counterinsurgency builds upon previous scholarship that has analyzed the humanitarian aid and peacebuilding sectors as increasingly complicit – if not interchangeable – with counterinsurgent aims (Moe, 2017; Turner, 2015). Our argument is also built in dialogue with literature that historicizes the professionalization of police alongside imperial counterinsurgency campaigns in the mid-20th century (Schrader, 2019) and which continues to recognize the relationship between policing and counterinsurgency in contemporary military operations (Kienscherf, 2019; Neocleous, 2011, 2020). Lastly, we are in conversation with scholarship that recognizes the coloniality of P/CVE and counterterrorism in Kenya (Oando, 2024; Oando and Achieng, 2021) and the ways in which P/CVE programming in Kenya overwhelmingly reflects the interests of international donors rather than local communities (Badurdeen, 2023). Recognizing P/CVE-as-counterinsurgency as a mode of governance, as ‘a form of politics in and of itself’ (Charbonneau, 2021: 1806), is critical in understanding the manifestations of police power which it both enables and requires.

The research for this article was conducted during two separate periods of field research: the first between January 2018 and February 2019, immediately following the DusitD2 complex attacks, and the second between September 2021 and June 2022. The first period of research took place in the scope of a non-governmental organization-funded project focused on women, peace, and security and P/CVE; insights regarding police violence and police reform were not the primary aim of the research but were nevertheless present in nearly every interview and focus group discussion. The second period of field research was conducted as part of a larger academic research project on P/CVE in Kenya and was sanctioned by a university ethics committee and a NACOSTI research license from the state of Kenya. In total, this article draws on qualitative data from approximately 65 interviews and informal talks with police officers, government officials, security personnel, civil society actors, and community activists in the areas of Nairobi, Kwale, Mombasa, Wajir, Garissa, and Isiolo. During both periods of research, we took care to build relationships with communities and security officials, employing feminist ethnographic methods to enable a deeper understanding of the context and our positionality within it. As two white women from the United States and Europe, respectively, our race, gender, and nationality enabled greater access to civil society spaces than we might have had otherwise. Civil society organizations often met us with hopes for access to funding that we did not possess. Navigating access in what is a deeply transactional research context required a sustained mindfulness of our privilege and power in the field as we endeavored to make our intentions transparent. In addition, given our respective backgrounds researching and serving in militaries, we were able to navigate more complex and sometimes hostile situations with security actors, situations that our positionality as white women both exacerbated but also alleviated. Ultimately, while all research participants gave informed consent for their quotes to be used, we did not record interviews and we chose to use pseudonyms and to withhold identifying data that could potentially put participants – or ourselves – at risk.

Based on this field research, this article theorizes discursive and material activities we understand to comprise policing – namely police reform and police brutality – and their relationship to P/CVE-as-counterinsurgency. The first section provides background to the evolution of Kenya’s counterterrorism agenda and the concurrent calls for police reform. We argue that the calls for police reform can never be anything more than rhetorical given that they occur within and alongside an agenda that simultaneously ensures the expansion of state and extralegal power. Further, we see police reform agendas as, in part, the result of normative compliance that occurs within the international community, whereby states must comply with particular security agendas or lose donor funding or face other material repercussions. The P/CVE industry and the initiatives to reform police and increase community engagement have offered the Kenyan government a means to ‘mimic’ internationally acceptable forms of governance as it attempts to offset the issue of police brutality with community-friendly policing, but also expand the power of the security state and enable the continuation of abject forms of violence.

The second section looks at this exact tension by analyzing a dialectic of policing practices in Kenya: police violence and police reform. The tension between extrajudicial killings, enforced disappearances, and other forms of violence that we term ‘abject coercion’ and the calls for police reform that enable community engagement, a form of ‘coercive compliance’, is understood as a dialectic rather than a paradox, particularly when considered in relationship to the international normative compliance of the P/CVE agenda. The final section connects the policing tactics of P/CVE-as-counterinsurgency to the colonial history of Kenya, drawing an analogy between the contemporary case of Mathare and Eastleigh, where extrajudicial killings are rampant and justified through racialized anti-terror rhetoric, and the British counterinsurgency campaign against the so-called Mau Mau insurgency. While killings within the informal settlement of Mathare and the neighborhood of Eastleigh and the brutal counterinsurgency waged against the Kikuyu population in the mid-20th century might seem disparate examples, we argue that the racial logic of colonial control found in British imperial policing is similarly present in current manifestations and policing of the ‘violent extremist’ or otherwise ‘suspect’ individual. Here, we do not mean to draw a comparison between the Mau Mau and Al-Shabaab, but rather to argue that the sediment of British colonial rule can be seen in the racialized logics that underwrite contemporary manifestations of security practices targeting the violent extremist.

## Counterterrorism, police reform, and the evolution of the P/CVE agenda in Kenya

Kenya is of central geopolitical importance to the global war on terror – a fact that both increases scrutiny of, but also contributes to, the rampant police brutality and human rights abuses perpetrated by security forces. Following the 1998 US Embassy bombings in Nairobi and Dar es Salaam and the 2002 attacks by al-Qaeda on the Israeli-owned Paradise Hotel near Mombasa and an Israeli airliner departing Mombasa’s Moi International Airport, Kenya has been under significant pressure to ramp up its counterterrorism measures to ensure stability in the Horn of Africa. While some scholars have assessed Kenya’s compliance with the international counterterrorism regime in the first decade of the global war on terror as weak (Whitaker, 2010), others argue that Kenya’s counterterrorism agenda is directly influenced by the international community and the United States in particular (Mogire and Agade, 2011; Prestholdt, 2011). The intersection of security and development and the perception of ungoverned or ‘undergoverned’ spaces as posing threats to the international liberal order squarely situated Kenya as a key state for Western counterterrorism (Bachmann, 2012). The United States Agency for International Development (USAID), for instance, was tasked with strengthening the counterterrorism capacities of Kenyan police in 2004 (Hills, 2006) and remains an active donor of P/CVE programming to date. Since 2002, the United States has trained hundreds of Kenyan security officials, many of whom have ended up in the Anti-Terrorism Police Unit (ATPU), which was formed in direct response to the attacks in Mombasa. These attacks put pressure on the Kenyan government to denounce terrorism and take decisive action; international travel advisories, including warnings from the US Embassy, significantly derailed Kenya’s tourist industry (Prestholdt, 2011). Kenya further cemented its role in the global war on terror with Operation Linda Nchi, the military invasion of Southern Somalia by the Kenya Defence Forces (KDF) in 2011 (Anderson and McKnight, 2015). While Kenya operated without the direct support or even approval of the United States and other Western allies, the KDF and the African Union Mission (AMISOM) has received ample logistics and financial support, equipment, and training from the United States since 2007 (Anderson and McKnight, 2015).^1^

At the same time Kenya’s counterterrorism strategy was evolving, police reform was simultaneously becoming a point of national focus, particularly in light of the post-election violence of 2007–2008, during which 405 people were shot dead by police. As legal scholars and human rights advocates Biegon and Songa (2018) point out, Kenya’s counterterrorism measures have had a deleterious effect on police reform, particularly the attempt to institutionalize rights-based policing (2018: 197–220). As they write, ‘Investment in police reform has focused almost entirely on enacting laws and establishing institutions. Little has been done to reform the culture of human rights violations and abuses which necessitate the reform in the first place’ (Biegon and Songa, 2018: 206). In Kenya, as in many countries the world over, the protection of human rights is treated as antithetical to or incommensurate with national security. This is clear in Kenya’s 2012 Prevention of Terrorism Act (POTA) and the 2014 Security Laws Amendment Act (SLAA), which further expanded the power of security and intelligence apparatuses and have been routinely used to perpetrate human rights abuses and surveil and target Muslim and ethnic Somali communities (Ogada, 2016; Republic of Kenya, 2008). While we do not dispute that the counterterrorism agenda has contributed to the inability to successfully implement police reform, we see the P/CVE agenda as another element that aids what Biegon and Songa (2018) call ‘reform speak’ – or, the rhetorical pandering to reform without the ultimate objective of substantive or meaningful transformation.

The P/CVE agenda appears to offer a solution to the incompatibility of Kenya’s counterterrorism agenda and its police reform agenda by complying with the international community’s shift toward prevention while still enabling police greater access to communities through programming that targets enhanced cooperation and greater trust. In 2016, Kenya was one of the first countries worldwide to adopt a National Strategy to Counter Violent Extremism (NSCVE), immediately following the United Nations’ adoption of the Plan of Action to Prevent Violent Extremism in 2015, which names police reform as an important pillar of prevention (Crisman et al., 2020; Sharamo and Mohamed, 2020). Heralded as a pioneer in the advancement of the P/CVE agenda, Kenya’s NSCVE de-emphasized the security tactics of military, police, and surveillance in favor of development, peacebuilding, and community resilience – an important rhetorical shift away from the hardened policing practices exacerbated by Kenya’s alliance with Western counterterrorist agendas. The 2016 NSCVE was seen as a needed corrective, by both the state and community organizations, to the ‘hard’ security approaches enacted by police and military. Anchored in eight pillars through which the ‘soft’ approaches to countering violent extremism can be actualized, the NSCVE emphasizes cooperation between state and non-state actors in the implementation of prevention measures intended to enable communities to ‘reject violent extremist ideologies and aims in order to shrink the pool of individuals whom terrorist groups can radicalize and recruit’ (Ogada, 2017: 1–2). The emphasis on state–civil cooperation is drawn from the 2015 UN Plan of Action to Prevent Violent Extremism, which also emphasizes the need to facilitate better engagement with communities through the adoption of

community-oriented policing models and programs that seek to solve local issues in partnership with the community and are firmly based on human rights so as to avoid putting community members at risk. This would increase public awareness and vigilance and improve police understanding and knowledge with regard to communities, thus enhancing their ability to be proactive and identify grievances and critical issues at an early stage. (UN General Assembly, 2015: 16)

Given the ‘trust deficit’ between police entities and the policed communities created by police brutality, enforced disappearances, and extrajudicial killings, police reform, community policing, and improving community and police relationships have been key priorities of the international P/CVE agenda. Beyond the moral and ethical considerations for ending police brutality, the logic of police reform is embedded in the prevention framework: if police are less violent and more trustworthy, the better the collaboration and cooperation will be with communities, and the more intelligence police will be able to access. Further, the deployment of P/CVE strategies by police is seen as critical, given that negative encounters with security institutions – such as being stopped and searched for no obvious reason, corruption, violent interactions including extrajudicial killings or enforced disappearances – often lead to grievances and frustration where the security sector itself becomes the source of instability or injustice (Holmes, 2017). The failure or deliberate denial of protection to certain groups or people can cause a profound animosity towards these institutions, which in literature on radicalization is defined as a contributing factor for people to become radicalized or to express sympathy towards violent groups and their objectives (Watanabe, 2018). The European Commission, for instance, deliberately lists community policing as a type of P/CVE intervention in its regional training curriculum on P/CVE. It encourages

police officers to be attentive to their citizens’ problems, be reliable and responsive, be honest when a job can or cannot be done, address the community with respect and treat all community members fairly. These practices also help to ensure that the behaviors of law enforcement do not inadvertently contribute to grievances that can fuel radicalization. (European Commission, 2020: 92)

P/CVE thus provides the Kenyan state a framework through which to comply with what sociologist Hajjar (2019) calls the ‘counterterrorism war paradigm’, which has resulted in the global dispersion of contested legal and policy measures but which also now focuses on prevention. The manifestation of this normative compliance with the international community is underwritten by, as feminist theorist Ahmed (2003) argues,

democratic norms of behavior and conduct, of what it means to be civil, a civil society, and a legitimate government. To be brought into the international civil society – that is, to be not named as a ‘rogue state’ or as part of ‘the axis of evil’ – others must ‘mimic’ these rules of conduct and forms of governance. (2003: 394)

The African continent in particular has been subjected to this normative compliance through describing states in pathological categories of failure, weakness, fragility and – fortified through the global war on terror – as security threats (Hagmann and Hoehne, 2009; Williams, 2006). Kenya’s timely adoption of the NSCVE and its increase in attention to police reform since the introduction of the global P/CVE agenda speaks to P/CVE as an international mode of governance, which compels states to both comply with the ‘counterterrorism war paradigm’ and the brutal policing that accompanies it, while simultaneously enacting police reform vis-a-vis community engagement. This tension cannot be dismissed as coincidental, but rather, we argue that these two modes of policing comprise a dialectic central to P/CVE-as-counterinsurgency. The calls for police reform neither negate nor deter the enactment of police violence; rather, they obscure and, arguably, sanction such violence. Ultimately, this twinning mode of controlling populations allows for the continuation of violent policing while projecting to the international community compliance with normative international agendas that center police reform as critical for good governance.

## Two sides of a coercive coin: The concurrent manifestation of police violence and police reform

Kenya’s adoption of a NSCVE and compliance with the international P/CVE agenda, which increased its attention to and resources for police reform, has resulted in two seemingly opposed outcomes: on the one hand, the P/CVE agenda has provided the police with better access to communities and the intelligence imagined to lie therein through community engagement programming, but it has also tacitly sanctioned the expansion of police brutality by widening the range of activities considered to be ‘violent extremism’. Brian, the executive director of a civil society organization in Mombasa that refused P/CVE funding (a rarity in Kenya), explained that P/CVE and the language of ‘violent extremism’ has broadened the scope of what might be considered a security threat and thus actionable by security forces:

For wearing a t-shirt, you could be arrested, for donning dreadlocks you could be locked up. And that means that we have subordinated the whole discourse of human rights that we have had since 1948. And in its place, we brought security to the fore. (Interview 13)

The well-documented risks posed by the P/CVE agenda to human rights, including freedom of expression (see Shanaah, 2023), can be read as tactics of abject coercion used by police to instill fear and force compliance among populations.

Other tactics of police violence used to control populations include the wanton destruction of property and terrorizing of villages. For instance, Marcus, who worked for a large international non-governmental organization (INGO) implementing P/CVE programming focused on community–police relations, spoke of the ‘massive deployments of security’, including the Kenyan Police Service (KPS) and the National Police along with the Anti-Terror Police Units, in areas such as Lamu, Garissa, and Mandera (Interview 1). Police, he explained, would come in following terrorist attacks and burn down entire markets or destroy livestock. ‘They are going for the heads’, he said, ‘not of the terror group but of the people’ (Interview 1). The total lack of trust and fear of the police leaves communities more susceptible to recruitment from the very non-state armed groups they are suspected of being a part of; as Marcus put it, they are caught ‘between the terror and the police’ (Interview 1). Joseph, the executive director of a peacebuilding NGO based in Nairobi, explained the high levels of ‘extrajudicial killings or mysterious disappearances’ lead communities to turn against the police (Interview 2). Police will go and raid communities, carrying out ‘heinous crackdowns’ as they ‘hunt’ for suspects, justifying the brutal tactics through claims to national security (Interview 2). In such an environment, Joseph explained, ‘trust is just impossible. Nobody can report to the police anymore, because nobody trusts them’ (Interview 2).

This absence of trust leads, of course, to silence. A governmental official from Isiolo also spoke to the silence created by fear: ‘You fear the terrorists, but you equally fear the government, so people just keep quiet’ (Interview 5). In Mombasa, a peacebuilder explained that the fear of speaking about violent extremism led to a hesitance to report, which in turn created what she called ‘a culture of silence’ (Interview 4). Fear of the police also impedes the abilities of human rights and peacebuilding organizations in their attempts to build better community–police relationships. As Joseph explained, with ‘projects like ours that have to work with the police, we become the immediate suspect’ (Interview 2). A member of the Police Reforms Working Group, an organization composed of 20 civil society organizations working toward police reform, commented on the importance of the network for the individual safety of its members:

There is strength in numbers and there is security in numbers. So even if they want to target one institution, the other institutions will be watching over them – it is watching over the human rights crusaders, that’s how we call them. (Interview 6)

Despite such attempts to self-protect against the police, community actors are acutely aware of the risks they face. As one activist put it:

I have to tell you, it’s difficult. Sometimes we try to push, but you realize that in the end of the day, you have a life to live . . . sometimes you don’t even know who is sitting next to you. At the end of the day, I’m just a human being. They can decide to abduct me. (Interview 7)

This ‘culture of silence’ created by the ‘trust deficit’ makes successful community engagement – a supposed cornerstone of P/CVE – virtually impossible.

The seemingly paradoxical manifestation of police violence next to policing reforms is perhaps most evident in the institution of Kenya’s National Police Service (NPS). Within this institution, we witnessed both the continuation of violent brutality against communities and the attempt by police to navigate calls for police reforms primarily through investing in community engagement. While Kenyans are wary of ‘the police’, which includes the wide array of security institutions that fall under the command of the NPS, there is specific apprehension regarding the Anti-Terror Police Unit (ATPU). The ATPU, a specialized unit within the Directorate of Criminal Investigation (DCI), is particularly infamous for using excessive force, extrajudicial killings, and torture. Described by one research participant as ‘a gang of criminals that work for the government’, the ATPU was formed in 2003 as a direct government measure to tackle terrorism. In 2022, the ATPU opened its Coast Regional Headquarters and Mombasa Police Station, funded by the UK at GBP 7 million a year. The joint initiative is said to ‘strengthen counterterrorist capacity within the criminal justice system, in line with international human rights standards’ and emphasizes ‘not only a strong security response, but also a holistic preventative effort that incorporates political, diplomatic and development responses’ (British High Commission Nairobi, 2022). Communities, however, greatly fear the ATPU given its violent tactics. Jon, a former member of the KDF, spoke bluntly about ‘badness’ of the police, particularly in comparison to the military, which, according to him, operates with more integrity (Interview 8). In Kenya, the aim of the ATPU is to obtain information and eliminate threat: they capture a person, interrogate the person, and kill or ‘disappear’ the person. The goal, according to Jon, ‘is that they [the population] are afraid’ (Interview 8). While many community activists remain hopeful that their local police services are reformable, and some have reported seeing improvements with increased police training and dialogue between officers and community members, such hope is non-existent when considering the ATPU.

While the ATPU yields tactics of fear and brutality, members of the KPS as well as the Administrative Police Service (APS) – police officers who live within communities – focus on community engagement and police/community initiatives, which aim to tackle the ‘trust deficit’. In 2003, the KPS changed its name from Kenyan Police *Force* to Kenyan Police *Service*. This rebranding was described by another government official of Isiolo as an attempt by the police ‘to be more or less customer friendly’ (Interview 9). Rather than relying on abject coercion through extrajudicial killings, enforced disappearances, or torture, officers of the KPS strive to be accepted within communities. Such acceptance, however, is greatly hindered by the nature of random police rotations, through which officers are transferred mere months after a new deployment. The local police who are tasked with fostering trust and building community relations thus lack the time, resources, and incentives to produce substantive reform. The result is superficial engagement with communities, who are primarily treated as intelligence sources from which to quickly extract potentially actionable information. The fact that one institution, the NPS, simultaneously calls for police reforms and invests in community engagement initiatives while continuing to support the ATPU’s excessive use of force, extortion, torture, and extrajudicial killings suggests that the tactics of police brutality and police reform are actually mutually reinforcing rather than mutually exclusive.

Indeed, calls for police reform and community engagement are, in part, directly related to the desire to obtain greater intelligence from communities. In instances where research participants did report improved relationships with the police through P/CVE initiatives – referring to more ‘cordial’ relationships, greater trust between the parties, and less aggressive behavior from the security sector more broadly – the exchange of information was always mentioned. The same government official from Isiolo as mentioned above, for instance, reported an improvement in the relationships between security actors and the community, which led directly to ‘the volunteering of information about terrorism, about recruitment. It’s for the exchange of information between the government, the agencies, and the people. It’s now very cordial . . . people feel free to share with the government’ (Interview 9). More respectful and less violent exchanges with communities not only decreases the likelihood of individuals turning toward non-stated armed groups based on grievances, but also increases the amount of security intelligence the police are able to gather from the population. As the government official said, it is the community that is ‘supposed to gather information, information that is good in terms of security aspects, and then pass it on to the police and the local administrators and chiefs’ (Interview 9). As a high-ranking police officer based in Nairobi articulated, the acquisition of intelligence is precisely the rationale for non-coercive, non-violent engagement with communities:

The main goal is to make sure that the ideology doesn’t infiltrate the population . . . The number one goal is that they don’t let the ideology and the terrorist in. And number two is, if they’re drawn into it, to de-radicalize them and bring them out. We do capacity building for our police officers: If the people feel that they are treated with injustice, they will never give information to the police. So, we have to have a discussion together which creates room for engagement. (Interview 10)

The concept of ‘room for engagement’ is a critical component of P/CVE-as-counterinsurgency: through ‘coercive compliance’, communities are forced to participate in their own policing. As in civil counterinsurgency, inoculating populations against the lure of insurgents, or, in this case, terrorists, is a first step to defeating the insurgency. However, if the ideology does manage to ‘infiltrate the population’, the next step would be to ‘de-radicalize them and bring them out’. Such a security approach is only ever possible, however, if the population is more convinced by the security forces and the government which they represent, than they are by the insurgents or terrorists. The abject coercion of police violence would therefore seem to foreclose any such possibility of engagement.

The seeming paradox of police brutality and police reform within the framework of P/CVE-as-counterinsurgency suggests an inability to detach policing from its history of imperial violence. The goal of successfully engaging the community is in line with pacification, a mode of counterinsurgency Schrader (2019) defines as ‘future-oriented, community-based, civilian-led proactive methods to prevent civil violence . . . [with] ongoing prevention as its goal. The most mobile aspects of pacification were not the most abjectly coercive but rather those that worked to further political participation and police-enforced rule of law’ (2019: 145). Despite calls for police reform, police in Kenya continue to enact ‘the most abjectly coercive’ modes of pacification, fostering distrust and contempt between communities and the police. P/CVE-as-counterinsurgency in Kenya comprises a dialectic of violence and reform, abject coercion and coercive compliance. P/CVE both enables police brutality by widening the scope of activities and individuals considered suspect but also relies upon the ability to successfully engage with communities in order to gain intelligence and ‘draw out’ potential insurgents. This dialectic, however, is not exclusively found within P/CVE but has its roots in imperial policing and counterinsurgency campaigns designed to both ‘civilize’ and ‘sanitize’.

## The sediment of racial and colonial logic in P/CVE-as-counterinsurgency

In January 2019, Elizabeth accompanied David, a social justice activist who specifically works on police violence, on a visit to Mathare, where he was born and raised. Mathare is an artificially constructed valley, carved out through the mining of quarry stones to obtain the material needed to build homes and buildings during Britain’s early colonial rule. Now, with a population density of more than one thousand residents per hectare and most residents earning less than USD 1 per day, Mathare faces, among other problems, limited access to basic services including clean water, poor sanitation, increased risk of accelerated spread of infectious disease, and low-quality housing (see United Nations Habitation, 2020). Police killings in Mathare, like in other informal settlements in Nairobi and Mombasa, are frequent and the perpetrators are rarely, if ever, held accountable. Police will kill peaceful protestors, individuals suspected of crimes ranging from petty theft to brewing illicit alcohol, as well as those suspected of being ‘radical’ or ‘violent extremists’. It is this latter category, ‘the violent extremist’ – a discursively and legally expanded version of the ‘the terrorist’ – that has come to serve as a catchall for those individuals deemed ungovernable, unworthy, lives feminist political theorist Judith Butler would call ‘ungrievable’ (Butler, 2010).

In a discussion of the ramifications of the P/CVE agenda in his neighborhood and on his work, David explained that the concept of violent extremism would be better understood as a social than a legal problem; such acts of extreme violence are, according to him, the direct result of the failure of the state to understand and provide for the people it governs. As David articulated, the contemporary figure of the ‘violent extremist’ is an individual marked as a threat to the state, whose rebellion or insolence poses a risk to governance. In particular, David posited that today’s ‘violent extremists are simply a reincarnation of the Mau Mau’, the Kikuyu anti-colonial fighters who rose up against British rule in the mid-20th century (Interview 17). Inspired by his analogy between the category of ‘violent extremist’ and the Mau Mau, we consider the role of policing in P/CVE-as-counterinsurgency as informed by – but not reducible to – counterinsurgent methods originating during the twilight of the British Empire. This analogy is particularly interesting given the supposed hearts-and-minds approach of British counterinsurgencies, which were guided by a doctrine of minimum force and emphasized non-military or non-kinetic solutions, much like P/CVE. Recent scholarship, however, has dispelled the idea that the British counterinsurgency in Kenya was anything other than brutally violent (Branch, 2010; Branch and Wood, 2010; Elkins, 2005). In this section, we theorize the sediment – and the sentiment – of the racialized counterinsurgency campaigns of British colonial rule in Kenya as clearly visible in the abject coercion and coercive compliance of the contemporary P/CVE agenda.

In response to rising militancy against the colonial state, primarily but not exclusively within the Kikuyu population, the colonial government declared a state of emergency in October 1952. For the next four years, colonial security forces waged a counterinsurgency against those named the Mau Mau, leaving tens if not hundreds of thousands of Kikuyu people dead, tortured, hanged, starved, displaced, and more than one million interned in detention camps. While the complete details of the counterinsurgency campaign are outside the scope of this article, drawing an analogy between the Mau Mau and the contemporary figure named ‘violent extremist’ allows for a theorization of the continued – albeit revised – role of racialization in the construction of individuals who are in need of either elimination or rehabilitation. Indeed, the Mau Mau were presented as ‘a barbaric, anti-European, and anti-Christian sect that had reverted to tactics of primitive terror to interrupt the British civilizing mission in Kenya’ (Elkins, 2005: xi). Newsinger (1981) writes that British authorities portrayed the Mau Mau through ‘racist caricature’ and the uprising as ‘a barbaric tribal response to the pressures of modernization, as a reversion to primitive superstition and blood-crazed savagery caused by the inability of the Africans to cope with the modern world’ (1981: 159). Such characterization enabled the British, as well as many Kenyans, to view the Mau Mau as terrorists, rather than, as historian Wa-Githumo (1991) writes, ‘a revolutionary and military response to the imperialists’ incursions, aggression, land expropriation, as well as the exploitation of the Africans’ natural and human resources’ (1991: 2).

Drawing an analogy between the Mau Mau and the contemporary category of ‘violent extremist’ – which can include, but is not reducible to Al-Shabaab^2^ – is not meant to collapse everything under an explanatory framework of colonialism. Rather, our theorization that the role of policing within P/CVE-as-counterinsurgency contains sediment of British colonial logic and imperial tactic is meant to draw attention to how processes of racialization – the categorization of individuals not necessarily along phenotypical lines but through modes of ascribing value that continues to privilege and protect certain lives over others – shift throughout time and alongside evolving geopolitical interests. As critical race theorists Daulatzai and Rana (2015) write, ‘“Terror” talk is the new race talk – the “terrorist” (or the “militant” or the “radical”) is the twenty-first-century way of saying “savage”’ (2015: 39). Contemporary incidences of police killings, carried out in response to activities that range from peaceful protests to suspected terrorist activity, can be read as the reanimation of imperial policing tactics through newly racialized registers. Kenya’s alliance with the United States in the global war on terror and against Al-Shabaab in Somalia in particular renders the ethnic Somali Muslim – particularly those that are refugees – living in Kenya a suspect category of individual. Much as the Mau Mau’s strong territorial claims and deep desire to defend their land rendered them a category of individual that had either to be killed or, if possible, rehabilitated and ‘civilized’ – what today might be referred to as ‘deradicalized’ – the contemporary violent extremist is imagined by the police as a similarly abject category of individual: to be feared, to be managed, or to be marked for elimination.

Such an analogy is perhaps nowhere more clear than in the parallels between Operation Anvil and Operation Usalama Watch. Carried out in April 1954, Operation Anvil was a plan to purge the ‘non-desirable elements of Nairobi, those seen as ethnically and ideologically affiliated to Mau Mau’ (Kimari, 2022: v). Tens of thousands of men, women, and children from across Nairobi, including large numbers from Mathare, were relocated to Kikuyu settlements or were interned in concentration camps. Many were killed or disappeared. The operation was a surprise; residents of Nairobi awoke to military vehicles with loudspeakers broadcasting instructions to pack one bag and leave home immediately. While the entire population was initially swept up, it was only Africans that were taken to holding areas, where their tribal affiliations were determined. Kikuyu, Embu, and Meru individuals were detained and processed according to a ‘pipeline’, which first classified suspects based on their level of risk and their likelihood of being receptive to rehabilitation.^3^ Those categorized as ‘black’ were seen as beyond redemption and sent to ‘special detention camps’. The operation was considered a ‘success’ by police and military standards: ‘Anvil had produced a great quantity of intelligence, the passive wing in Nairobi had been broken, and the forest gangs had been further isolated’ (Crow, 1971: 151). Nairobi was ‘clean’.

Almost 60 years later in April 2014, Kenyan security forces carried out Operation Usalama Watch, a counterterror operation focused primarily on Mombasa town and the Nairobi neighborhood of Eastleigh. Eastleigh is often referred to as ‘little Mogadishu’ due to its inhabitants from Somali and Kenyan Somali communities (Herz, 2008). The objective was to ‘flush out Al-Shabaab adherents to deter terrorism’, with Eastleigh being of primary importance given its high population of ethnic Somalis (Wairuri, 2018). Over the course of months, the operation included demolitions, arrests, police raids, forced relocations and deportation of refugees, as well as the internment of hundreds of people under inhumane conditions in the Kasarani stadium (Balakian, 2016; Glück, 2017). In public communication, the operation was lauded as a direct response to Al-Shabaab attacks that happened in the previous months, namely the Westgate shopping mall attack in September 2013. Internally, however, the operation came to be known as Operation Sanitise Eastleigh (Institutional Policing Oversight Authority [IPOA], 2014). The idea of Eastleigh and Mombasa town as in need of ‘cleansing’ was rooted in the belief that radicalization and violent extremism propagates in such spaces, which are characterized as chaotic and dangerous (Glück, 2017; Herz, 2008).

The criminalization and unlawful detainment or execution of particular individuals based on racial, ethnic, or religious categories is, of course, not exclusive to a post-9/11 world order in which state powers have grossly expanded in the name of ‘prevention’ (see for instance McQuade, 2021). The signifiers have shifted but the rationale remains the same: just as the Mau Mau were targeted through tactics of imperial policing to quell the rebellion and maintain British colonial rule, ethnic Somalis and Muslims are now targeted through similar tactics of policing, detention, and execution. In our field research, numerous participants spoke of the problem of profiling ethnic Somali and Muslim populations, particularly refugees, as terrorists. In Isiolo, for instance, during a roundtable discussion with youth leaders, we learned of how the police label youth as radicals, as violent extremists, or as Al-Shabaab to such an extent that young men feel they have no other choice than to become the terrorist they are already assumed to be. This type of prophecy is decried by youth leaders, who fear the despair of a population that feels it has no other choice, that their fate has already been decided by police (Interview 14).

The fear of being associated with terrorism based on one’s race, ethnicity, dress, or religion is prevalent throughout Kenya, particularly in areas with Muslim and ethnic Somali majorities. A religious leader in Nairobi spoke of the ‘thin line between terrorism, religion, and communities in Africa’. He explained, ‘If I am Swahili and I am a Muslim, there is a likelihood that if anything happens around terrorism, I become the first suspect . . . I am a potential suspect, just because of my personal structure’ (Interview 15). Similarly, a community leader from Lamu, a predominantly Muslim area, stated that he had been detained on suspicion of terrorist activity numerous times, without evidence: ‘Why? Because I just have a long beard and I look like a Somali’ (Interview 16). Another individual living in Nairobi described the associations that come with being a Muslim in Kenya:

It’s a big problem for most of us, if you are a Muslim by faith, you are already a terrorist, so the only thing that they are trying to do, is to make sure they can either arrest you or follow you. If you are a Muslim and you walk into a police station in Kisumu, the police say we are in danger here [because of the person walking into the station who is regarded as a threat]. So that’s very bad, it’s profiling an individual based on what they see. (Interview 6)

The rampant Islamophobia, racial and religious profiling, and the projection of a discourse of violent extremism onto Muslim populations leads, of course, to a deep distrust of the police by Muslim communities. The idea that ‘if you are a Muslim by faith, you are already a terrorist’ results in an unbridgeable gap between communities and the police. A county official from Wajir explained that as long as the police continue to kill with impunity, there would be no ‘winning’ against terror. ‘We’re not trying to win the hearts and minds of the people’, they said. ‘We’re not trying to build trust and confidence in the problem. We have lost the public. You’re better off with Al-Shabaab than with Kenyan security agencies’ (Interview 18).

The racialized categorization of Muslim and ethnic Somali populations as, to use Heath-Kelly’s (2013: 397) phrase, ‘at risk of becoming risky’ can be read as a contemporary manifestation of colonial-era concerns with unmanageable populations. Indeed, since the launch of the US-led global war on terror, scholars have critiqued the global counterterrorism agenda as a re-entrenchment of the ‘civilizing missions’ central to colonialism. Mark Neocleous, for instance, argues that at the contemporary intersection of the police and war is an internationally governing ideology underwritten by colonial logic of civilizing populations. As Neocleous (2011) states, the global war on terror is ‘the violent fabrication of world order in exactly the way that the original police power was the violent fabrication of social order. The war on terror, as international politics, is a form of police; civilization’s return, writ large’ (2011: 156). The brutal repression of the Mau Mau insurgency offers a powerful analogy to understand the contemporary enactment of P/CVE-as-counterinsurgency, which simultaneously enacts abject violence against marginalized Muslim and ethnic Somali communities while promising reform and the space for community engagement. This, however, is not a paradox but an international and local mode of governance dependent on a dialectic of violence and rehabilitation, of abject coercion and coercive compliance, that is integral to P/CVE-as-counterinsurgency.

## Conclusion

P/CVE, as a policy and practice, is agile, capable of being many things: a tool for community engagement to overcome the ‘trust deficit’ between communities and police; a mode to gather human intelligence for security purposes; an industry through which donors can allocate funds toward ‘meaningful’ programming within multiple sectors including development, peacebuilding, and security; and an opportunity to comply with calls for police reform with minimal impact on actual police practice. As a security architecture, a funding stream, and a set of normative commitments to aligning against terrorism, P/CVE provides the Kenyan police with the justification to commit extralegal acts of violence – what we have called ‘abject coercion’. P/CVE also provides a framework through which the Kenyan government can, at least rhetorically, call for police reform and enable the police to more successfully engage communities and gather intelligence – tactics of ‘coercive compliance’. However, the P/CVE agenda’s codification of the logic of prevention – that terrorism or violent extremism or insurgency is always about to erupt – into national and international security architectures, renders actual substantive reform both unlikely and unattractive. What calls for police reform do enable is a way to measure states’ compliance with international norms – a way for Western powers to govern their allies. Reformism can be read as type of security mimicry whereby human rights are lauded as important and yet the security objectives upheld ensure that the protection of rights or the guarantee of basic human needs are never the state’s primary concern.

We have argued that abject coercion and coercive compliance are a dialectic integral to counterinsurgency, two seemingly opposed forces that work in tandem, tethering principles and practices of violence to rehabilitation and reform. This analytical purchase of this dialectic is not limited to the security architecture of P/CVE and counterterrorism in Kenya. Rather, exemplified through P/CVE, we demonstrate that the comprehension and implementation of contemporary understandings of ‘security’ are deeply informed by twinning modes of violence and community engagement. The doctrinal and operational shift in peacekeeping towards peace-enforcement missions, for instance, contains the dialectic of abject coercion and coercive compliance integral to counterinsurgency. Peacekeeping operations in the Democratic Republic of Congo, Mali, and the Central African Republic, which Karlsrud (2015: 41) described as tantamount to ‘us[ing] peace operations to wage war’, focused on civilian engagement and human intelligence gathering while simultaneously targeting and eliminating actors deemed threats to keeping the peace. Manifestations of the abject coercion/coercive compliance dialectic can be found throughout contemporary security contexts, particularly those that rhetorically espouse commitments to peace, human rights, limited use of force, or reform without substantive enactment of such principles.

Ultimately, by insisting that P/CVE *is* counterinsurgency, we have offered a framework to critically examine the very foundations upon which today’s counterterrorism architecture is built, questioning the sedimented racial and colonial logics and further theorizing counterinsurgency as a mode of governance. In Kenya, the mutually constituting operations of police violence and police reform comprise the central functioning of P/CVE-as-counterinsurgency, a modality of governance that contains colonial sediment and reproduces racialized categories of individuals to be civilized, saved, rehabilitated, or eliminated.

## Notes

1. Journalist Nick Turse reports that AMISOM, which protected the US-supported government in Mogadishu, received at least USD 650 million with an additional 100 million since 2011. Turse also reports that US Special Operations have played ‘a crucial role in infrastructure investments for the Kenyan Special Operations Regiment and especially in the establishment of the Kenyan Ranger School’. (Turse, 2015: 25, 54).

2. The Somali-based militant group, Al-Shabaab, is considered the most serious threat to the stability of the Horn of Africa as it controls many rural areas of Somalia and is responsible for frequent attacks and killings in the region. Al-Shabaab is a Salafist ‘jihadi’ Islamic movement which first emerged as an urban militia in defense of the Islamic Courts Union (ICU) in Mogadishu, and later underwent several transformations to wage an insurgency in Kenya and Southern Somalia (Anderson and McKnight, 2015).

3. For a discussion of ‘rehabilitation’ as a euphemism for civilizing, see Elkins, 2005: 91–120.
